# Clients’ satisfaction with preventive services for malaria during pregnancy in Anambra state, Nigeria

**DOI:** 10.1186/s12889-020-09767-2

**Published:** 2020-11-04

**Authors:** Emmanuel Chijioke Obagha, IkeOluwakpo Ajayi, Gobir A. Abdullahi, Chukwuma David Umeokonkwo

**Affiliations:** 1 Nigerian Field Epidemiology and Laboratory Training Programme, Abuja, Nigeria; 2 Epidemiology Unit, Public Health Department, Anambra State Ministry of Health, Awka, Anambra State Nigeria; 3 University of Ibadan, Oyo State, Nigeria; 4 Ahmadu Bello University Zaria, Kaduna State, Nigeria; 5 Department of Community Medicine, Alex Ekwueme Federal University Teaching Hospital Abakiliki, Ebonyi State, Nigeria

**Keywords:** Client satisfaction, Knowledge, Malaria in pregnancy, Anambra state, Nigeria

## Abstract

**Background:**

Burden of Malaria in pregnancy (MIP) is still high despite availability of proven cost-effective interventions. Considerable progress has been made on improving antenatal attendance, but MIP preventive services utilization remains low. Factors responsible for this include dissatisfaction with the services provided. We assessed clients’ satisfaction with preventative services for malaria during pregnancy delivered at antenatal clinics (ANC) in Anambra State Nigeria.

**Method:**

We conducted a cross-sectional study among 284 pregnant women attending ANC using multistage sampling technique. Pre-tested semi-structured interviewer-administered questionnaire was used to collect information on socio-demographics, knowledge of malaria in pregnancy services and satisfaction with services. Responses to questions on satisfaction was on a 5-point Likert scale. A cut off of ≥75% of composite score was used to classify respondents as satisfied. For knowledge, every correct answer was scored 1 and incorrect 0; ≥75% of the composite score was graded as good knowledge. Chi square and logistic regression were used to test for association between client satisfaction and independent variables.

**Results:**

The mean age of participants is 28 years±4.4 years. Overall, 62.2% were satisfied with quality of preventive services for malaria during pregnancy. However, 64.8 and 57.8% were dissatisfied with cost of healthcare and interpersonal relationship with the health workers (HWs). Majority of the respondents (88.73%) had poor knowledge of malaria preventive services during pregnancy. Type of facility (Adjusted odds ratio [aOR] = 2.11; 95%CI: 1.20–3.71) and knowledge (aOR: 0.41; 95%CI: 0.18–0.90) were independently associated with satisfaction with interpersonal relationship. Type of facility (aOR: 0.47; 95%CI: 0.27–0.80) and employment status (aOR: 3.07; 95%CI: 1.39–6.74) were also independently associated with satisfaction with cost of healthcare.

**Conclusion:**

A fair proportion of respondents were satisfied with the preventive services for malaria during pregnancy provided even though most were dissatisfied with the cost of assessing care and interpersonal relationship with health workers. Uninterrupted availability of long lasting insecticide treated bed nets and intermittent preventive treatment for malaria at all health facilities, subsidized cost of malaria-related commodities, and incentives for good customer satisfaction ratings may remediate the described barriers to seeking preventative care for malaria during pregnancy.

**Supplementary Information:**

**Supplementary information** accompanies this paper at 10.1186/s12889-020-09767-2.

## Background

Burden of Malaria in pregnancy (MIP) is still high despite availability of proven cost-effective interventions [1]. Globally approximately 25 million pregnant women are at risk of infection by *Plasmodium falciparum* annually [2]. “In Africa, 30 million women living in malaria-endemic areas become pregnant each year. For these women, malaria is a threat both to themselves and to their babies, with up to 200 000 new-born die each year as a result of malaria in pregnancy” [3]. Nigeria accounted for 27% of global malaria cases, the highest in the world [4]. In 2004, the Federal Government of Nigeria adopted control and prevention of malaria in pregnancy strategy as a component of Focused Antenatal Care with a view to drastically reduce the burden of malaria in the country [5].

Considerable progress has been made in improving antenatal attendance but MIP services utilization remains low [6]. Factors responsible for this include dissatisfaction with the services provided [7]. Satisfaction with care, a patient based assessment, is one of the outcome measures in assessing quality of care. The recommended intervention strategies for preventing malaria during pregnancy in Nigeria are the use of intermittent preventive treatment (IPT) with sulfadoxine-pyrimethamine under direct supervision of skilled health providers and use of insecticide-treated bed nets [5].

Improving the quality of care has been shown to increase uptake of services and reduce the number of adverse maternal health outcomes. WHO opined that the wellbeing of mothers and babies depends on an operational continuum of care and accessibility of high quality care during pregnancy [8]. A study conducted by Majrooh et al. on coverage and quality of antenatal care provided at Primary Health Care Facilities in the Punjab Province of Pakistan found a drop in antenatal attendance from 55.9 and 32.9% between first and subsequent attendance due to poor quality of services [9]. In many African countries, the coverage of malaria in pregnancy preventive services is low and reports have mentioned utilisation of care as one of the major obstacles [7]. Fagbamigbe and Idumudia in assessing barriers to antenatal care use in Nigeria with evidence from non-users highlighted issues that have to do with satisfaction with quality of care as the major barriers [10].

A number of authors in Africa have studied the satisfaction of the women with preventive services for malaria during pregnancy they receive [11–13]. However, little have been documented in Nigeria. This study was therefore conducted to assess client satisfaction with the quality of preventive services for malaria during pregnancy delivered at antenatal clinics in selected health facilities in Anambra State, Nigeria.

## Methods

Anambra State has 21 Local Government Areas (LGA) and 75% of them are classified as rural. There are public and private owned health facilities in the state. The State Ministry of Health manages the public health facilities. These public health facilities comprise of 560 primary healthcare centres, 36 secondary health facilities and 2 tertiary health facilities. There are 305 primary health facilities and 1394 secondary facilities owned and managed by individuals and private organizations. Most public health facilities are not functional leading to high dependence on the private facilities. No record was found on the employee strength of the private facilities but there are 85 doctors and 613 nurses in the public owned facilities. Most of the free commodities like Rapid Diagnostic (RDT Kits), Long Lasting Insecticidal Treated Net (LLIN), anti- malaria drugs are distributed mostly to the public owned facilities through the state central medical store and the state malaria elimination programme.

A cross-sectional study conducted between May and August 2018. We recruited 284 antenatal attendees, who have completed at least one ANC visit using a three-stage probability sampling technique. In stage one; we selected one LGA each from the three senatorial districts in the state by simple random sampling. In stage two; health facilities in the selected LGAs were stratified into primary health care (PHC) and secondary facilities. The secondary health facilities are further stratified into public secondary and private secondary health facilities. One facility was selected from each of the three strata (primary, public secondary and private secondary health facilities) by simple random sampling giving a total of nine facilities. Number of participants per facility was proportionally allocated using average antenatal attendance per month from daily attendance register. Among the selected PHCs, the average monthly attendance ranged from 18 to 50 clients, among the private secondary facilities it ranged from 64 to 150 clients while for public secondary facilities it ranged from 30 to 300 clients. In the third stage; systematic random sampling was used to select the participants. Sample size of 284 was estimated using formula for single proportion [14]

$$ n=\frac{Z_{\alpha}^2P\left(1-P\right)}{d^2} $$

p was estimated using satisfaction level of pregnant women towards antenatal care at Ibadan southwest Nigeria (81.1%) [15]. Pregnant women attending antenatal clinics in Anambra State, who consented to participate, were included in the study whereas; those who presented for the first time in the antenatal clinic in the index pregnancy were excluded.

Data was collected using pre-tested semi-structured interviewer administered questionnaire developed for this study. The questionnaire had three sections: socio-demographics, knowledge and satisfaction questions which was divided into five domains: - process of care (7 items), interpersonal relationship/communication (7 items), hospital/clinic environment (8 items), accessibility (2 items), and cost of healthcare (2 items). The part on knowledge was designed after literature search while the section on satisfaction was adapted from Patient Satisfaction Questionnaire, a standardized questionnaire [16], and modified it to suit our study. The patient satisfaction questionnaire had earlier been validated in the same state with Cronbach alpha of 0.78 [17]. Translation to native languages was done in cases where the respondents do not speak English. Back-translation was also done to avoid ambiguity and ensure uniformity of understanding. Questionnaire was pre-tested on 30 respondents at a facility not included in the study site and data was collected electronically using Open Data Kit (ODK). After pre-testing the questionnaire, corrections were made. The questions contained positively and negatively framed questions to limit information bias, which will skew the data.

The data was collected by three research assistants who are trained nurses working as disease surveillance and notification officers in the state. The research assistants were trained for 2 days. Interviews were conducted as exit interviews.

We exported the data to excel and analyzed using epi-info 7.2.1. We ensured that the data was complete during the collection stage by building in check codes into the electronic questionnaire and making all the questions required. Knowledge of malaria in pregnancy preventive services was tested using 11 questions on malaria in pregnancy. Responses to knowledge questions were scored one every correct answer and zero for every wrong answer. Composite score of ≥75% was graded as good knowledge otherwise knowledge was graded as poor. Satisfaction questions on satisfaction were on a 5-point Likert scale with score values ranging from 1 (strongly disagree) to 5 (strongly agree). Then a cut off of ≥75% of composite score was classified as satisfied, otherwise unsatisfied. Univariate analysis was done to generate frequencies and proportions. Chi square was used to test for association between client satisfaction and the sociodemographic characteristics. The factors that were had *p* value of less than 0.1 were modelled in a logistic regression to at 5% level of significance to identify the predictors of satisfaction.

Ethical approval with ref. number COOUTH/CMAC/RP/Vol.1/0029 was obtained from ethical committee of Chief Odumegwu Ojukwu University Teaching Hospital. A written informed consent form was obtained. Confidentiality of the information collected was maintained. The data was only used for research purpose and access was only granted to appropriated personnel involved in the study.

## Results

A total of 284 participants completed the study. The mean age of the participants was 28.0 ± 4.4 years and most of them 192 (67.61%) were in the 25–34 years age group (Table 1). Majority 259 (98.85%) were Igbos and 259 (98.85%) were married. Two hundred and fifty-two (88.73%) of respondents had poor knowledge of malaria in pregnancy preventive services.

**Table 1:** Sociodemographic characteristics of respondents

Variable	Frequency (%)
**Age group (years)**	
15–24	73 (25.70)
25–34	192 (67.61)
35–44	17 (5.99)
45–54	2 (0.70)
**Marital status**	
Single	8 (2.82)
Married	276 (97.18)
**Education**	
None	4 (1.41)
Primary	15 (5.28)
Secondary	185 (65.14)
Tertiary	80 (28.17)
**Parity**	
Primipara	78 (27.46)
Multipara	196 (69.01)
Grand multipara	10 (3.52)
**Type of facility**	
PHC	32 (11.27)
Private Secondary	88 (30.99)
Public Secondary	164 (57.75)
**Employment Status**	
Unemployed	22 (7.75)
Employed	262 (92.25)

Overall, 188 (62.2%) of the participants were satisfied with the with malaria preventive services they received (Table 2). The satisfaction level however varied in the different satisfaction domains. Most respondents were not satisfied with the interpersonal relationship/communication (57.75%) and cost of healthcare (64.79%, Table 2).

**Table 2:** Satisfaction level with domains of client satisfaction among pregnant women at antenatal clinics, Anambra state, 2018

Domain of satisfaction	Satisfied n (%)	Unsatisfied n (%)
Process of care	267 (94.01)	17 (5.99)
Interpersonal relationship/ communication	120 (42.25)	164 (57.75)
Hospital environment	187 (65.85)	97 (34.15)
Accessibility	178 (62.68)	106 (37.32)
Cost of healthcare	100 (35.21)	184 (64.79)
Overall satisfaction	188 (62.20%)	96 (33.80%)

Employment status, type of facility and knowledge of malaria preventive services were associated with interpersonal relationship/communication domain of quality of care (Table 3). These variables were entered into logistic regression model (Table 4), only type of facility (Adjusted odds ratio [aOR] 0.41, 95% CI 0.18–0.90, p 0.02) and knowledge (aOR 2.11, 95% CI 1.20–3.71, *p* < 0.01) remained significantly associated with satisfaction in interpersonal relationship/communication.

**Table 3:** Factors associated with satisfaction in interpersonal relationship domain among pregnant women in Anambra State, 2018

Variable	Satisfaction Level		OR (95% CI)	*p*-value
	Satisfied (%)	Unsatisfied (%)		
**Age**				
< 25	42 (57.53)	31 (42.47)	0.99 (0.58–1.69)	1.00
≥ 25	122 (57.82)	89 (42.18)		
**Employment Status**				
Employed	116 (34.57)	146 (65.43)	0.28 (0.09–0.85)	0.02
Unemployed	4 (18.18)	18 (81.82)		
**Parity**				
Multi	85 (41.26)	105 (58.74)	1.15 (0.69, 1.96)	0.58
Primi	35 (44.87)	43 (55.13)		
**Education**				
Secondary	82 (44.32)	103 (55.68)	0.63 (0.37–1.11)	0.18
Tertiary	27 (33.75)	53 (66.25)		
**Type of facility attended**				
Private secondary	26 (29.55)	62 (70.45)	2.0 (1.19, 3.57)	< 0.01
Public secondary	76 (46.34)	88 (53.66)		
**Knowledge**				
Poor	101 (40.08)	151 (59.92)	2.18 (1.03, 4.62)	0.03
Good	19 (59.38)	13 (40.63)

**Table 4:** Predictors of satisfaction in interpersonal/ communication domain with malaria in pregnancy services among women attending ANC in Anambra State, 2018

Factors	aOR (95% CI)	***p***-value
**Type of facility**		
Private secondary	1	
Public secondary	2.11 (1.20–3.71)	< 0.01
**Employment Status**		
Unemployed	1	
Employed	0.35 (1.11–1.08)	0.07
**Knowledge**		
Poor	1	
Good	0.41 (0.18–0.90)	0.02

Education, type of facility, and knowledge were found to be associated with cost of healthcare satisfaction domain (Table 5). However, only type of facility (aOR 0.47, 95% CI 0.27–0.80, p < 0.01) and knowledge (aOR 3.07, 95% CI 1.39–6.74, p < 0.01) remained significantly associated with satisfaction with cost of healthcare domain after logistic regression (Table 6).

**Table 5:** Factors associated with satisfaction in cost of healthcare domain among pregnant women in Anambra State, 2018

Variable	Satisfaction Level		OR (95%CI)	*p*-value
	Satisfied (%)	Unsatisfied (%)		
**Age**				
< 25	24 (32.88)	49 (67.12)	1	
≥ 25	76 (36.02)	135 (63.98)	1.15 (0.65–2.02)	0.63
**Employment Status**				
Employed	93 (35.50)	169 (65.50)	1.18 (0.46–3.00)	0.15
Unemployed	7 (31.82)	15 (68.18)	1	
**Parity**				
Multi	71 (34.47)	135 (65.530	1	
Primi	29 (37.18)	49 (62.82)	1.13 (0.65–1.93)	0.67
**Education**				
Secondary	72 (38.92)	113 (61.08)	0.56 (0.31–2.20)	0.05
Tertiary	21 (26.25)	59 (73.75)	1	
**Type of facility attended**				
Private secondary	43 (48.86)	45 (51.14)	1	
Public secondary	50 (30.49)	114 (69.51)	0.46 (0.27–0.78)	< 0.01
**Knowledge**				
Good	19 (59.38)	13 (40.63)	3.09 (1.45–6.55)	< 0.01
Poor	81 (32.14)	171 (67.86)	1

**Table 6:** Predictors of satisfaction in cost of healthcare domain with malaria in pregnancy services among women attending ANC in Anambra State, 2018

Factors	aOR (95% CI)	***p***-value
**Type of facility**		
Private secondary	1	
Public secondary	0.47 (0.27–0.80)	< 0.01
**Knowledge**		
Poor	1	
Good	3.07 (1.39–6.74)	< 0.01

## Discussion

High proportion of women had poor knowledge about malaria in pregnancy preventive services though high proportions of them were satisfied with the services they are receiving. However, most of them were not satisfied with the interpersonal relationship with the health service providers and cost of healthcare services they received. Type of facility attended, and employment status remained as predictors of satisfaction in interpersonal and cost of services received.

The low level of knowledge found in this study could be due to the poor interpersonal relationship between the patients and the health workers. This contrasts with the study conducted in Ebonyi State where they found high level of knowledge among pregnant women [18]. This was also inverse with the findings of a study conducted in the Federal Capital Territory, Northern Nigeria which reported that over 70% had good knowledge [19] though the study was conducted among attendees in a tertiary institution. A dissimilar knowledge level was also found in Calabar, Cross River State in a study [20]. The difference in the findings of these studies with our study could be because they assessed general knowledge on malaria but our study looked at only knowledge of preventive services, which could also be why our study had similar findings with the study conducted in Lagos, South western Nigeria, where they found knowledge of malaria prevention among pregnant women and care givers and care givers of under-five.

The study found that just above half of the respondents to be satisfied with preventive services for malaria during pregnancy but many respondents were not satisfied with the interpersonal relationship and cost of healthcare. The non-satisfaction with interpersonal relationship could be due to the small number of health workers in the state, leading to high volume of patients for available personnel. This could shorten the time for the client health provider interaction or even lead to reduce quality of service provided. The interpersonal relationship is a major factor in a client experience in hospital service provision.

The high level of dissatisfaction with cost of healthcare could be attributed to the high cost of services due to non-availability of LLIN and IPTp provided by government. Generally, government provides these preventive interventions free through the public health facilities and some selected private health facilities. These services are often not available due to stock out or inefficiency the distribution network. The non-availability mean that clients will have to pay for these services often out of pocket. Without limited health insurance cover and out of pocket spending, it could easily tip pregnant women and their families into catastrophic health spending and hence the dissatisfaction. The relationship between cost of services and satisfaction with health services have earlier been reported [17].

There is an overall high satisfaction with the process of care, environment and accessibility. This could be because patients attend ANC care where they are convinced is good for them. The findings from study conducted in a cottage hospital in Port Harcourt that assessed satisfaction with care in relation to antenatal care contrasted with our study [21]. In this study, 94% of the respondents were satisfied. The lowest satisfaction score was in area of medical consultation, which is comparable with findings from our study, even though this study was conducted in only one hospital. Oladapo et al. in assessing quality of antenatal care in primary health facilities in southwest Nigeria got high satisfaction of 81.1% which also contrasts with our study [22]. Nwaeze et al. at Ibadan got a satisfaction score of 81.1% among pregnant women attending ANC at a public hospital [15]. The difference in our findings could be because their study was conducted in only one hospital. In Ethiopia, Ejigu et al. (2013), in studying Quality of antenatal care services at public health facilities of Bahir-Dar special zone, Northwest Ethiopia found that 47.7% of women were not satisfied. The findings from this study are comparable to the findings in our study.

The positive association found between private health facility and satisfaction in interpersonal domain could be explained by the fact that doctors in private facilities take special care in order not to lose their clients but those in public facilities have a care free attitude because their salaries does not depend on the number of clients available. This association is the reverse when it comes to the relationship between private facilities and cost of healthcare. Possible explanation of this is that private hospitals are generally more expensive than the public hospitals and they do not benefit most times from free commodities supplied to the public facilities. Poor knowledge was also positively associated with satisfaction in the cost of healthcare. Clients are possibly going to be satisfied with what they are offered if they have poor idea of what they are supposed to get. This contrasts with the study conducted by Do et al. in Kenya and Namibia, where they assessed satisfaction with antenatal care. In the study most demographic characteristics were significantly associated with satisfaction [23]. The difference could be due to geographical location and cultural differences. The difference could also be because we are looking at malaria in pregnancy services, which is just a part of what is offered in antenatal clinic. Our finding was similar to finding in a study on satisfaction among pregnant women towards antenatal care in public and private care clinics in Khartoum; attending private health facility was positively associated with satisfaction [24].

Information bias is one of the major limitations associated with satisfaction surveys. Dunsch et al. in identifying pitfalls of client satisfaction found about 40% drop in satisfaction if questionnaire was administered at home rather than as client exit. They also found a difference in satisfaction level when questionnaires contain only positively framed questions in relation to positive and negative framed questions [25]. In this study, we tried to reduce this bias by framing positive and negative questions but we collected the data as exit interview due to the cost of visiting each patients home to collect data. Another limitation was finding studies that have worked on satisfaction with preventive services provided for malaria during pregnancy, so we compared our study with finding from studies conducted in quality of antenatal care in the same target population.

## Conclusions

High proportion of respondents had poor knowledge of malaria in pregnancy. A fair proportion of respondents were satisfied with the MIP services provided even though most were unsatisfied with the cost of assessing care and interpersonal relationship with health workers.

Type of facility attended, and employment status were associated with predictors of satisfaction with interpersonal relationship / communication and cost domain of satisfaction. We recommend that the state government through the ministry of health and health managers should subsidize the cost of preventive services for Malaria in pregnancy. Efforts at improving the quality of client health provider interaction could help in improve client satisfaction. This could also help improve the amount information the clients get about their health condition hence ultimately improving their knowledge.

## Supplementary Information

**Additional file 1.** Study questionnaire. Blank questionnaire containing all questions designed for this study.

## Data Availability

The data generated and used for this research is available from the corresponding author on reasonable request.

## Abbreviations

ANC: Antenatal care
aOR: Adjusted Odds Ratio
FGD: Focus Group Discussion
IPT: Intermittent Preventive Treatment
LGA: Local Government Area
LLIN: Long Lasting Insecticide Treated Net
MIP: Malaria in Pregnancy
