# Supplementary material for: Clients’ satisfaction with preventive services for malaria during pregnancy in Anambra state, Nigeria
Source: BMC Public Health. 2020 Nov 4;20:1660. doi: 10.1186/s12889-020-09767-2 (PMC7641832; doi:10.1186/s12889-020-09767-2)
Supplement: Supplementary file 1 — Additional file 1. Study questionnaire. Blank questionnaire containing all questions designed for this study. [file 12889_2020_9767_MOESM1_ESM.pdf]

Appendix_IV

Questionnaire on Client Satisfaction with the Quality of Malaria in Pregnancy Preventive Services Delivered at Antenatal Clinics in Selected Health Facilities In Anambra State, Nigeria

I, Obagha Chijioke Emmanuel, wish to kindly request your voluntary participation by providing honest answers to the following questions which are aimed at providing evidence base for your satisfaction with malaria in pregnancy services delivered during antenatal services in Anambra State. The information obtained will be used to Plan strategies to improve the quality of services provided. Your kind cooperation in supplying correct information to the questions below will be highly appreciated. All information provided by you would be strictly confidential.Should I continue?

- ◯ yes
- ◯ no

date

yyyy-mm-dd

Questionnaire number

2. Interviewers name

3. Interviewers number

Capture location
capture less than 20m

latitude (x.y °)

____

longitude (x.y °)

____

altitude (m)

____

accuracy (m)

____

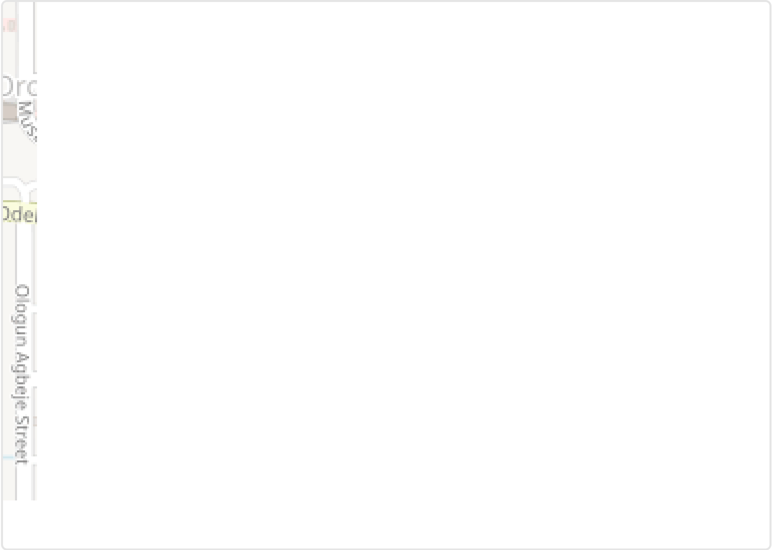

Socio-demographics

4.How old are you?

____

5. What is the sex of the patient?

- ○ Male
- ○ Female

6. What is your marital status?

- ○ Married
- ○ Single
- ○ Divorced
- ○ Separated
- ○ Widowed

7.What is your religion?

- ○ Christianity
- ○ Islam
- ○ Traditional
- ○ Other

8.Please Specify

---

9. What is your ethnic group

- ◯ Igbo
- ◯ Hausa
- ◯ Yoruba
- ◯ Other

10. Please Specify

---

11. What is your highest education level?

- ◯ No formal Education
- ◯ Primary Education
- ◯ Secondary Education
- ◯ Tertiary Education
- ◯ Other

11b. Please Specify

---

12. What is your occupation

- ◯ Civil servant
- ◯ House Wife
- ◯ Self employed

13. How many times have you been pregnant?

____

14. Type of Facility attended

- ◯ PHC
- ◯ Public Secondary
- ◯ Private Secondary

KNOWLEDGE OF RESPONDENTS ON MALARIA IN PREGNANCY PREVENTIVE SERVICES

15.Have you heard of malaria before?

- ◯ yes
- ◯ no

16.What was your source of information?
select all that apply

- ☐ Radio
- ☐ TV
- ☐ Health Personnel
- ☐ Internet
- ☐ Friends
- ☐ Other

17. Please Specify

18.What are the causes of malaria?
select all that apply

- ☐ Witchcraft
- ☐ Mosquito
- ☐ Taking too much oily food
- ☐ Stress
- ☐ Other

19. Please Specify

20.What are the symptoms of malaria?

21.How is malaria transmitted?
select all that apply

- ☐ Drinking contaminated water
- ☐ Mosquito bite
- ☐ Insect bite
- ☐ Aerosol
- ☐ Other

22.Please Specify

23.Pregnant women and Children are most at risk of malaria infection?

- ◯ yes
- ◯ No
- ◯ Don't Know

24.Unborn child is protected by the placenta from the harmful effects of malaria?

- ◯ yes
- ◯ No
- ◯ Don't Know

25.Which of these malaria preventive services are offered at ANC?
select all that apply

- ☐ Use of insecticide-treated nets (ITNs)
- ☐ Intermittent preventive treatment (IPT) with sulfadoxine-pyrimethamine (SP)
- ☐ Indoor residual spraying (IRS)
- ☐ Other

26. Please Specify

27.Pregnant women should seek services for prevention of malaria from:
select all that apply

- ☐ In the community
- ☐ Through traditional healers
- ☐ During regularly scheduled antenatal care visits
- ☐ Other

28.Please Specify

29.The World Health Organization recommends that pregnant women in areas of moderate-to-high malaria transmission take IPT with SP:

- ◯ At the beginning of pregnancy
- ◯ At least two times during pregnancy, after quickening
- ◯ At each scheduled antenatal care visit, starting the first dose as early as possible during the 2nd trimester of gestation

30.SP should not be taken by pregnant woman who take?

- ◯ Folic acid
- ◯ Are HIV positive and taking Cotrimoxazole
- ◯ Sleep under ITN
- ◯ Don't Know

31. The last dose of IPTp with SP can be administered up to the time of delivery, without safety concerns

- ◯ yes
- ◯ No
- ◯ Don't Know

32.Malaria test is not recommended before treating pregnant women showing signs and symptoms of illness

- ◯ yes
- ◯ No
- ◯ Don't Know

33.Pregnant women should not taking folic acid for one week after taking SP

- ◯ yes
- ◯ No
- ◯ Don't Know

CLIENT SATISFACTION WITH MALARIA IN PREGNANCY PREVENTIVE SERVICES DELIVERED DURING ANTENATAL CLINICS

Process of Healthcare

34. I spent enough time with the healthcare providers on this visit

- ◯ Strongly Agree
- ◯ Agree
- ◯ Indifferent
- ◯ Disagree
- ◯ Strongly Disagree

34b. On the average how many minutes did you spend with the healthcare provider

____

35.I waited for a very long time before I could see the healthcare provider on this visit

- Strongly Agree
- Agree
- Indifferent
- Disagree
- Strongly Disagree

35b. How many minutes did you wait before seeing the healthcare provider

____

36.I have confidence and trust in the doctors and nurses

- Strongly Agree
- Agree
- Indifferent
- Disagree
- Strongly Disagree

37.I was not given the opportunity to ask questions

- Strongly Agree
- Agree
- Indifferent
- Disagree
- Strongly Disagree

38.I was adviced on how to prevent malaria in pregnancy

- Strongly Agree
- Agree
- Indifferent
- Disagree
- Strongly Disagree

39.Sp is always available in this hospital

- ◯ Strongly Agree
- ◯ Agree
- ◯ Indifferent
- ◯ Disagree
- ◯ Strongly Disagree

39b. I have been given Sp during this pregnancy

- ◯ yes
- ◯ No
- ◯ Don't Know

39c. How many times

____

40c. How old is your pregnancy

____

40. LLIN is not available in this hospital

- ◯ Strongly Agree
- ◯ Agree
- ◯ Indifferent
- ◯ Disagree
- ◯ Strongly Disagree

40b. I was given LLIN during my first ANC visit

- ◯ yes
- ◯ No
- ◯ Don't Know

40c. How much did you pay for it?

____

Health workers Communication

41. I was not counselled properly on IPTp

- Strongly Agree
- Agree
- Indifferent
- Disagree
- Strongly Disagree

42.I was properly counselled on LLIN

- Strongly Agree
- Agree
- Indifferent
- Disagree
- Strongly Disagree

43.Provider counselling on nutrition was poor

- Strongly Agree
- Agree
- Indifferent
- Disagree
- Strongly Disagree

44.Provider listened and understood what was on my mind

- Strongly Agree
- Agree
- Indifferent
- Disagree
- Strongly Disagree

45.I was not involved in making decisions

- Strongly Agree
- Agree
- Indifferent
- Disagree
- Strongly Disagree

46. I sensed other patients could listen in when I was talking to the doctor

- Strongly Agree
- Agree
- Indifferent
- Disagree
- Strongly Disagree

47.The hospital staff do not treat me with respect and courtesy

- Strongly Agree
- Agree
- Indifferent
- Disagree
- Strongly Disagree

Environment

48.The clinic building is in good condition

- Strongly Agree
- Agree
- Indifferent
- Disagree
- Strongly Disagree

49.The clinic and its surroundings are dirty

- Strongly Agree
- Agree
- Indifferent
- Disagree
- Strongly Disagree

50.There are no toilets for patients in this clinic

- Strongly Agree
- Agree
- Indifferent
- Disagree
- Strongly Disagree

51. The clinic has enough consultation rooms

- ◯ Strongly Agree
- ◯ Agree
- ◯ Indifferent
- ◯ Disagree
- ◯ Strongly Disagree

51b. How many doctors stay in one consulting room

____

52. There are no benches for patients to sit while waiting to be seen by health worker

- ◯ Strongly Agree
- ◯ Agree
- ◯ Indifferent
- ◯ Disagree
- ◯ Strongly Disagree

53. The water in this facility is dirty

- ◯ Strongly Agree
- ◯ Agree
- ◯ Indifferent
- ◯ Disagree
- ◯ Strongly Disagree

54.There is clean water for IPTp intake

- ◯ Strongly Agree
- ◯ Agree
- ◯ Indifferent
- ◯ Disagree
- ◯ Strongly Disagree

55.There is no Laboratory in this facility

- Strongly Agree
- Agree
- Indifferent
- Disagree
- Strongly Disagree

Accesibility

56. The hospital is far from my house

- Strongly Agree
- Agree
- Indifferent
- Disagree
- Strongly Disagree

57. The money I spend to get to the hospital is much

- Strongly Agree
- Agree
- Indifferent
- Disagree
- Strongly Disagree

57b. How much do you spend on transport going to the hospital on each visit

____

57c. Are there hospitals closer to your house than this hospital

- yes
- No
- Don't Know

57d. Why don't you go to the hospitals? Are they public or private?

____

Financial Aspect

58.Cost of malaria drugs is cheap

- Strongly Agree
- Agree
- Indifferent
- Disagree
- Strongly Disagree

58. How much do you pay for antimalarial drugs?

____

59.Cost of Laboratory is expensive

- Strongly Agree
- Agree
- Indifferent
- Disagree
- Strongly Disagree

59. How much do you spend on malaria test

____

Overall Impression

60.I was not happy with the care receievd

- yes
- No
- Don't Know

61.Would you recommend this hospital to friends/family

- yes
- no
